# Multiplexed bovine milk oligosaccharide analysis with aminoxy tandem mass tags

**DOI:** 10.1371/journal.pone.0196513

**Published:** 2018-04-26

**Authors:** Randall C. Robinson, Nina Aagaard Poulsen, Daniela Barile

**Affiliations:** 1 Department of Food Science and Technology, University of California, Davis, Davis, California, United States of America; 2 Department of Food Science, Aarhus University, Tjele, Denmark; 3 Foods for Health Institute, University of California, Davis, Davis, California, United States of America; National Cancer Institute at Frederick, UNITED STATES

## Abstract

Milk oligosaccharides (OS) are a key factor that influences the infant gut microbial composition, and their importance in promoting healthy infant development and disease prevention is becoming increasingly apparent. Investigating the structures, properties, and sources of these compounds requires a host of complementary analytical techniques. Relative compound quantification by mass spectral analysis of isobarically labeled samples is a relatively new technique that has been used mainly in the proteomics field. Glycomics applications have so far focused on analysis of protein-linked glycans, while analysis of free milk OS has previously been conducted only on analytical standards. In this paper, we extend the use of isobaric glycan tags to the analysis of bovine milk OS by presenting a method for separation of labeled OS on a porous graphitized carbon liquid chromatographic column with subsequent analysis by quadrupole time-of-flight tandem mass spectrometry. Abundances for 15 OS extracted from mature bovine milk were measured, with replicate injections providing coefficients of variation below 15% for most OS. Isobaric labeling improved ionization efficiency for low-abundance, high-molecular weight fucosylated OS, which are known to exist in bovine milk but have been only sporadically reported in the literature. We compared the abundances of four fucosylated OS in milk from Holstein and Jersey cattle and found that three of the compounds were more abundant in Jersey milk, which is in general agreement with a previous study. This novel method represents an advancement in our ability to characterize milk OS and provides the advantages associated with isobaric labeling, including reduced instrumental analysis time and increased analyte ionization efficiency. This improved ability to measure differences in bioactive OS abundances in large datasets will facilitate exploration of OS from all food sources for the purpose of developing health-guiding products for infants, immune-compromised elderly, and the population at large.

## Introduction

Milk oligosaccharides (OS) have been intensely researched in the past two decades due to their multitude of bioactivities. Experimental evidence demonstrates that milk OS can act as prebiotics [1–3], prevent severe diarrhea [4, 5] and necrotizing entercolitis in infants [6], modulate glycan expression on intestinal cells [7], and act as decoys to prevent pathogen binding [1]. The prebiotic function of OS is theorized to provide human milk with the key ability to selectively modulate the gut microbiota of infants [8], and these compounds are believed to make a significant contribution to healthy infant development. The health benefits associated with milk OS have begun to motivate development of large-scale recovery techniques for the dairy industry, which would allow more widespread consumption of the typically underutilized OS in processing streams [9, 10]. Growing interest in milk OS, their *in vivo* functions, and opportunities for their utilization has created a need for efficient detection, characterization and quantification strategies.

**Fig 1:**
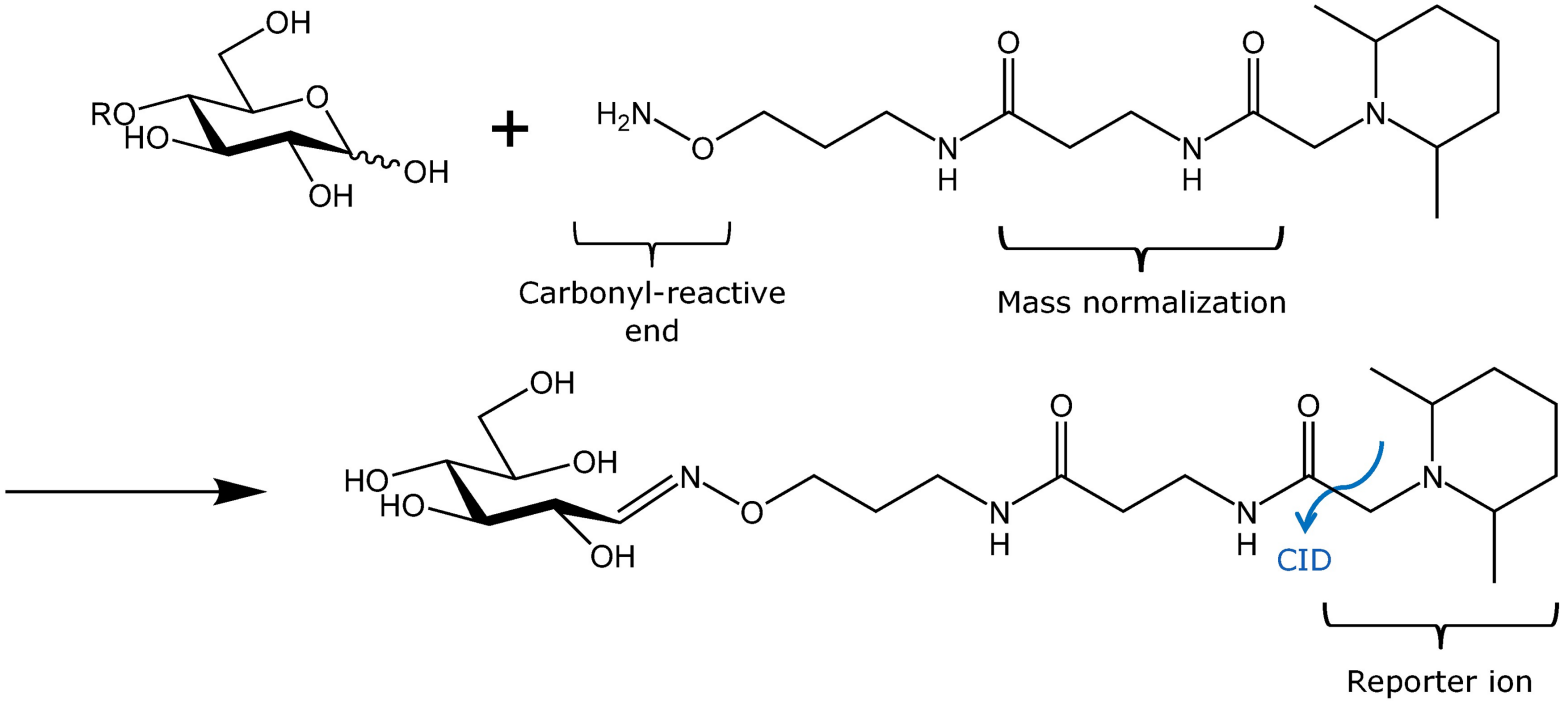
Tandem mass tag structure and labeling reaction. Using collision-induced dissociation, tags fragment as shown by the blue line above.

Qualitative and compositional analysis of OS mixtures is often achieved using high-performance liquid chromatography (LC) coupled to mass spectrometry (MS) [11, 12]. High-resolution MS instruments enable the types of monosaccharide residues (hexose, pentose, N-acetylhexosamine, etc.) in an OS structure to be identified, and the sensitivity of the technique allows for accurate relative quantification of a large number of OS in a single run. Although OS can be analyzed by LC-MS in their native state, chemical modification is often advantageous to avoid peak splitting from anomeric mixtures or to improve ionization. Common glycan modification techniques include reduction by NaBH_4_ [13–15], permethylation [16–18], and reducing end derivatization by reductive amination or hydrazone formation [19]. Reducing-end modification can also be used for the purpose of stable-isotope [20–22] and isobaric [23] labeling. Recently, a series of carbonyl-reactive isobaric tags (aminoxy tandem mass tags, or aminoxyTMTs) have been developed for glycan derivatization (Fig 1) [24, 25], which allow sets of samples to be multiplexed prior to LC-MS analysis. These tags function similarly to the isobaric tags used for peptide analysis [26–28] by bonding covalently with the analyte and generating reporter ions upon tandem-MS (MS/MS) fragmentation that serve as a basis for relative quantification. Each tag contains a unique arrangement of ^13^C and ^15^N isotopes, which causes each reporter ion variant to generate a distinct mass spectral peak when a set of multiplexed samples is analyzed during MS/MS experiments.

Application of isobaric labeling to OS analysis offers several advantages, primarily relating to throughput. LC gradients for milk OS analysis are typically in the range of 45–65 minutes per sample [29–31], which leads to long total runtimes when analyzing large sample sets. Analysis of multiplexed sample sets decreases this runtime without eliminating the chromatographic separation that is needed for isomer differentiation. The reduction in instrumental analysis time is advantageous both for the sake of efficiency and for minimizing the opportunity for drifts in instrument performance during the analysis of a sample set. Comparison of OS abundances within a multiplexed set also has the inherent advantage of eliminating the possibility for OS abundance measurements to be influenced by variations in ionization efficiency between runs. Furthermore, researchers have noted an increase in the ionization efficiency of aminoxyTMT-labeled vs. native glycans as a result of the tertiary amine in the tag structure [25], which can aid in detection of low-abundance compounds.

Methods using aminoxyTMTs for analysis of glycans covalently bound to proteins through asparagine residues (N-glycans) in human blood serum have been published recently and have demonstrated the accuracy and precision of the technique for quantitative measurements [25, 32]. Like released N-glycans, milk OS contain a single reducing end that serves as a site for aminoxyTMT labeling. Since TMT-based abundance measurements rely on generation of reporter ions through tandem fragmentation, the collision energy used for MS/MS fragmentation is a crucial component of these methods that must be carefully validated for the analytes under investigation. In this paper, we extend the use of aminoxyTMTs to the analysis of free OS by designing an optimized LC-MS/MS method for analysis of the OS in bovine milk and characterizing its accuracy, precision, and reproducibility. The technique is then applied to identify differences between cattle breeds in the abundance of fucosylated milk OS, a class of OS which is typically challenging to detect in mature bovine milk.

## Materials and methods

### Materials

Acetonitrile (ACN) (Optima LC/MS), methanol (Optima LC/MS), chloroform (Optima), and formic acid (FA) (Optima LC/MS) were purchased from Fisher Scientific (Fair Lawn, NJ, USA). Trifluoroacetic acid (TFA) was from Sigma-Aldrich (St. Louis, MO, USA). Ethanol (200 proof) was from Koptec (King of Prussia, PA, USA). C18 and porous graphitized carbon (PGC) solid phase extraction (SPE) microplates were purchased from Glygen (Columbia, MD, USA). Oasis HLB 60 mg SPE cartridges were from Waters (Milford, MA, USA). AminoxyTMT isobaric label reagents were from ThermoFisher Scientific (Waltham, MA, USA). The oligosaccharide standards 3’-sialyllactose (3’-SL), 6’-sialyllactose (6’-SL), lacto-N-tetraose, and lacto-N-hexaose were purchased from V-Labs (now Dextra Laboratories Ltd., Reading, UK). The OS standard GalNAc(α1–3)Gal(β1–4)Glc was from Dextra Laboratories Ltd. (Reading, UK). Raw milk used for collision energy optimization, method validation, and comparison of fucosylated OS abundances was collected under the Danish-Swedish Milk Genomics Initiative. This initiative collected morning milk samples from healthy Danish Holstein and Danish Jersey cows in midlactation within first to third parity. The complete sample collection process has been previously described in detail elsewhere [33]. Frozen sample aliquots were kept in storage at Aarhus University (Tjele, Denmark) and were shipped on dry ice to the University of California, Davis for this study. A standardized preparation of bovine milk OS powder [34], enriched in high-molecular weight fucosylated OS, was used as the internal standard mixture for comparison of fucosylated OS production between Holstein and Jersey cows.

### OS extraction

OS were extracted from bovine milk as described previously [35], with some modifications. Milk aliquots of 400 μL were combined with an equal volume of Milli-Q water and centrifuged at 4,000 × *g* for 30 min at 4 °C. The skim milk was transferred to a new tube and mixed with two volumes cold (-30 °C) ethanol, and the solution was placed at -30 °C for a minimum of 1 hour to precipitate intact proteins. The solution was then centrifuged at 4,000 ×*g* for 30 min at 4 °C, and the precipitated protein was discarded. The solution was dried by centrifugal evaporation (Genevac MiVac Quattro concentrator, Genevac Ltd., Ipswitch, England) and re-dissolved in 300 μL Milli-Q water for SPE purification.

The OS were purified by microplate C18 SPE to eliminate residual lipids and peptides. The solid phase was activated with ACN and equilibrated with water. Samples were loaded, and the solid phase was then washed with three column volumes (600 μL) Milli-Q water. The OS-containing eluate was collected and adjusted to a solvent composition of 2% ACN/0.1% TFA for subsequent lactose removal by microplate graphitized carbon SPE. The SPE wells were activated with 80% ACN/0.1% TFA and equilibrated with 2% ACN/0.1% TFA. After sample loading, lactose and salts were washed from the solid phase with 6 column volumes (1.2 mL) 2% ACN/0.1% TFA, and the OS were then eluted with 40% ACN/0.1% TFA.

### Isobaric labeling

Extracted OS and analytical standards were labeled with aminoxyTMTs according to instructions provided by the manufacturer. Briefly, each vial of isobaric label reagent was dissolved in 95% methanol/0.1% acetic acid, and the solution was transferred into tubes containing the dried standards or samples. The solutions were shaken for 10 minutes at 23 °C, then dried by centrifugal evaporation. 200 μL 95% methanol was added to each tube, and the tubes were shaken for 10 minutes at 23 °C. After drying, 100 μL 10% acetone was added, and the tubes were shaken for 10 minutes at 23 °C. The solutions were combined to form the desired multiplexed sets, dried, re-dissolved in 200 μL 50% ACN, and purified by SPE using Waters Oasis HLB cartridges. The cartridges were activated by washing the sorbent subsequently with 3 mL 95% ACN, 1 mL 50% ACN, and 3 mL 95% ACN. The labeled OS were diluted into 3 mL 95% ACN and loaded onto the column. Excess labeling reagent was washed from the cartridge with 6 mL 95% ACN, and the glycans were then eluted with 2 mL 50% ACN. For method development and optimization, analytical standards and OS extracted from a single milk sample were derivatized with a pair of label reagents in a 2:1 ratio using the above procedure.

### Comparison of fucosylated OS production in Holstein and Jersey cows

To demonstrate the utility of this novel workflow, we extracted OS from milk of the two major milk-producing cattle breeds, Holstein and Jersey (10 samples per breed). Using the above protocol, the milk samples were isobarically labeled, and a relative quantification of the high-molecular weight fucosylated OS was performed to investigate differences between the breeds in the abundance of these recently-identified compounds. Since this sample set could not be multiplexed into a single vial, we designed a technique by which the samples could be combined into a set of four vials and the reporter ion signals normalized to a common set of internal standards. OS collected from a standardized preparation of bovine milk OS powder [34] were extracted by PGC SPE as a large batch, labeled with the TMT^6^-126 reagent, and spiked into each multiplexed set as an internal standard mixture. Oligosaccharides from the bovine milk samples were labeled with the remaining (TMT^6^-127 through 131) labels and combined into four vials, each containing five samples.

### LC-MS/MS analysis of isobarically-labeled OS

Labeled analytical standards were dissolved in 3% ACN/0.1% FA prior to analysis. Samples were dissolved in 3% ACN and passed through 0.2 μm polyethersulfone filters (Agilent Technologies, Santa Clara, CA). LC-MS/MS analysis was performed with an Agilent 6520 Accurate-Mass Q-TOF LC/MS instrument equipped with a Chip Cube high performance liquid chromatography interface (Agilent Technologies, Santa Clara, CA, USA). Labeled OS were separated on a nano-LC PGC chip, containing a 40 nL enrichment column and a 75 μm x 43 mm analytical column, packed with 5 μm particles of 250Å pore size. The enrichment column was loaded with a capillary pump operating at a flow rate of 4 μL/min, and separation on the analytical column was achieved with a nanopump solvent flow of 0.3 μL/min. The mobile phase solvents were 3% ACN/0.1% FA (A), and 89.9% ACN/0.1% FA (B). The analytical and enrichment columns were equilibrated with 100% A, and a 65-minute gradient was used for chromatographic separation. The gradient was ramped from 4 to 20.6% B from 0 to 23 min, 20.6 to 50% B from 23 to 30 min, 50 to 100% B from 30 to 35 min, held at 100% B from 35 to 50 min, then lowered from 100 to 0% B from 50 to 50.1 min.

Mass spectra were collected in positive mode with scan ranges of 400 to 2500 (MS) and 100 to 2500 (MS/MS). Scan rates were two spectra/s for MS scans and one spectrum/s for MS/MS scans. An in-house library of bovine milk OS masses partially assembled from the literature [15, 31] was entered into the acquisition software as a list for targeted fragmentation. The five most abundant precursors in each MS scan matching to the targeted list were fragmented, with a quadrupole isolation window of ~4 m/z. A minimum precursor threshold of 5,000 ion counts/spectrum was set to ensure substantial reporter ion abundance in the MS/MS scans. Collision-induced dissociation (CID) was performed using nitrogen as the collision gas. CID energies were optimized to maximize the reporter ion signals generated by the isobaric labels and were specified separately for ions with charges 1–3 using the linear equation energy (V) = slope*((m/z)/100) + intercept. Slope and intercept values were as follows: 1+ ions: slope = 5.5, intercept = 11; 2+ ions: slope = 6, intercept = 0.16; 3+ ions: slope = 1, intercept = 35. Capillary voltage was varied from 1850 to 1875 V as needed to maintain a stable spray. Drying gas flow was 5 L/min at 350 °C. In-run calibration was performed using infused calibrant ions with m/z values of 922.009798 and 1221.990637. Data was stored in centroid mode.

### LC-MS analysis of underivatized OS

To evaluate the performance of the aminoxyTMT reagent in this novel application, samples used for method development were also analyzed in their underivatized state using an established LC-MS method [36] and the same instrumental setup described above. Briefly, after OS extraction, samples were dissolved in Milli-Q water and loaded onto the nano-LC chip, which was operated at flow rates of 4 μL/min (enrichment column) and 0.3 μL/min (analytical column). A 65-minute gradient for the analytical column was ramped from 0 to 16% B from 2.5 to 20 min, 16 to 44% B from 20 to 30 min, 44 to 100% B from 30 to 35 min, held at 100% B until 45 min, and then held at 0% B from 45.01 to 65 min.

Mass spectra were collected in positive mode over a scan range of 400 to 2500 m/z at a rate of two spectra/s. The drying gas was held at 350 °C with a flow of 5 L/min. In-run mass calibration was performed with infused calibrant ions of m/z 922.009798 and 1221.990637.

### Identification and relative quantification of isobarically labeled OS

The presence of each OS structure in each sample set was confirmed by examination of the MS/MS spectra using Agilent MassHunter B.06.00 (Agilent Technologies, Santa Clara, CA). For relative quantification, raw data was exported to .mzData format with MassHunter and then imported into SimGlycan Enterprise Edition 5.42 (PREMIER Biosoft, Palo Alto, CA) [37]. The OS which had been confirmed by MS/MS were entered into the SimGlycan server as a custom library, and the software then identified these OS in the data files by matching precursor mass and retention time using the “High Throughput Search and Score” feature. Precursor ion and reporter ion m/z tolerances were set to 10 ppm and 0.025 Da, respectively. All scans collected for each sample were used in the high-throughput search. For each OS, the reporter ion abundances from all the MS/MS spectra were summed, and the ratios of these sums were calculated. The reporter ion abundances for the Holstein vs Jersey OS comparison were normalized to the TMT^6^-126 signal that originated from the OS extracted in batch and spiked as an internal standard into each vial (illustrated in S1 Fig). The SimGlycan output for all analyses conducted in the study is provided as S1–S6 Tables. Unpaired, two-tailed t-tests for the Holstein vs. Jersey milk OS comparison were performed in Microsoft Excel.

## Results

### Method optimization with analytical standards

**Fig 2:**
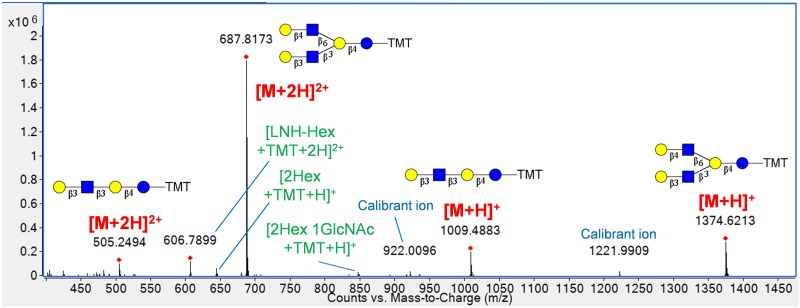
ESI mass spectrum of lacto-N-tetraose and lacto-N-hexaose standards mixed in a 3:2 molar ratio. The compounds ionized as hydrogen ion adducts and were observed as singly- and doubly-charged ions. In-source fragments are annotated in green.

A mixture of four OS normally found in bovine milk (3’-SL, 6’-SL, lacto-N-tetraose, and lacto-N-hexaose) [15, 38] was labeled in a 2:1 ratio with the TMT^6^-126 and TMT^6^-127 reagents. Upon LC-MS/MS analysis, the electrospray ionization (ESI) mass spectra showed that the OS standards ionized as hydrogen adducts with a mixture of 1+ and 2+ charges, as shown in Fig 2 for lacto-N-tetraose and lacto-N-hexaose. Sodium and potassium adducts were completely absent, providing low-complexity spectra. Although N-glycan hydrogen-ion adducts sometimes provide insufficient reporter ion abundance upon fragmentation [32], reporter ions in this case were abundant enough for successful quantification of all four OS standards. As shown in Fig 2, the OS experienced a minimal amount of in-source fragmentation. This fragmentation is not expected to affect relative quantification accuracy, as it occurs within the glycan structure and should therefore be experienced to an equivalent degree among all the isobaric label variants. The experimental ratios of the four standards measured from a single LC-MS/MS run are shown in Table 1. S2 Fig provides a sample MS/MS spectrum of each standard.

**Table 1:** Measured relative abundances from a single injection of OS standards labeled in a 2:1 ratio.

Oligosaccharide standard	Experimental ratio (126:127)	Observed m/z values
3’-SL	2: 1.14	935.4506 [M+H]^+^ 468.2289 [M+2H]^2+^
6’-SL	2: 1.22	935.4506 [M+H]^+^ 468.2289 [M+2H]^2+^
Lacto-N-tetraose	2: 1.16	1009.4874 [M+H]^+^ 505.2473 [M+2H]^2+^
Lacto-N-hexaose	2: 1.08	1374.6196 [M+H]^+^ 687.8134 [M+2H]^2+^

3’-SL, 3’-sialyllactose; 6’-SL, 6’-sialyllactose

**Fig 3:**
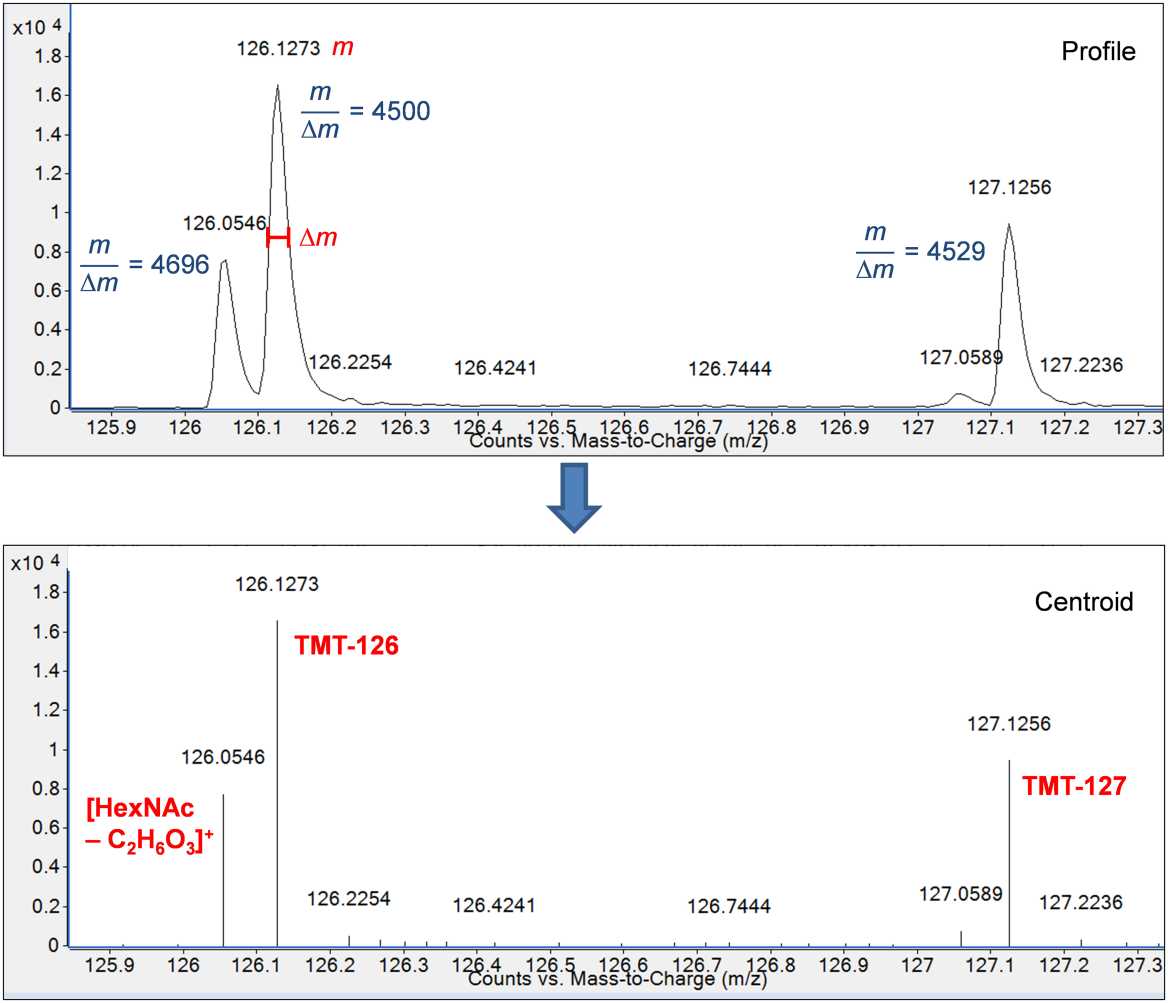
MS/MS spectrum of lacto-N-tetraose in profile (upper) and centroid (lower) formats. Resolution, defined as the quotient of m/z and peak width, or m/Δm, of the TMT^6^-126 reporter ion and the HexNAc oxonium ion at m/z 126.055 are sufficient for successful differentiation of the two peaks. The instrument provides near baseline separation of the ions in the profile data and allows successful conversion to centroid format.

A high proportion of milk OS contain N-acetylhexosamine (HexNAc) residues [15, 31], which produce characteristic oxonium ions during fragmentation [32, 39]. One of these oxonium ions appears at m/z 126.054 and requires a minimum resolution of ~2000 to be differentiated from the TMT^6^-126 reporter ion at m/z 126.1277. Successful differentiation of this oxonium ion from the TMT^6^-126 signal is a prerequisite for TMT quantification of HexNAc-containing glycans. We have measured resolutions above 4,000 for the oxonium and TMT^6^-126 reporter ion and have confirmed that the distinct peaks are preserved upon conversion to the centroid data format (Fig 3). This information ensures that the oxonium ion signal will not overlap with the TMT^6^-126 reporter ion and generate erroneous relative abundance measurements.

### Method validation with extracted milk OS

OS from a single milk sample were extracted and split into a 2:1 ratio prior to labeling to provide a simulated pair of multiplexed samples for further method development and validation. Analytical standards are unavailable for the majority of bovine milk OS, and so the collision energies for these OS were optimized using the extracted sample. A total of 15 OS were identified and quantified with the optimized method, as shown in Table 2. Four replicate injections provided coefficients of variation (CVs) below 15% for 11 of the 15 OS. Dynamic exclusion was disabled for all sample runs, allowing multiple MS/MS spectra to be collected for each compound.

**Table 2:** Measured relative abundances of 15 milk oligosaccharides identified from a single sample, labeled with two isobaric label variants in a 2:1 ratio. The listed ratios represent the average of four repeat injections of the same sample. Oligosaccharides are represented by their monosaccharide compositions, denoted as Hex_HexNAc_Fuc_NeuAc_NeuGc.

Oligosaccharide	TMT^6^-130 ion abundance	TMT^6^-131 ion abundance	TMT^6^-131 standard deviation	TMT^6^-131 coefficient of variation
2_0_0_1_0 (3’-SL)	2	1.12	0.02	1.4%
2_0_0_1_0 (6’-SL)	2	1.35	0.15	11.2%
2_0_0_2_0	2	1.32	0.29	22.1%
2_1_0_0_0	2	1.32	0.02	1.7%
3_2_0_0_0	2	1.19	0.02	1.8%
3_3_1_0_0	2	1.06	0.18	17.4%
3_6_1_0_0	2	1.04	0.06	5.4%
4_1_0_0_0	2	1.16	0.01	1.0%
4_1_0_1_0	2	1.27	0.03	2.0%
4_2_0_0_0 (LNH)	2	1.12	0.04	3.4%
4_4_1_0_0*	2	1.18	0.37	31.5%
4_5_1_0_0	2	1.01	0.21	21.2%
5_2_0_0_0	2	1.00	0.08	8.4%
5_4_0_0_0	2	1.11	0.03	3.1%
5_4_1_0_0	2	1.09	0.05	4.5%

3’-SL, 3’-sialyllactose; 6’-SL, 6’-sialyllactose; LNH, lacto-N-hexaose

*Only identified from 2 of 4 injections

**Fig 4:**
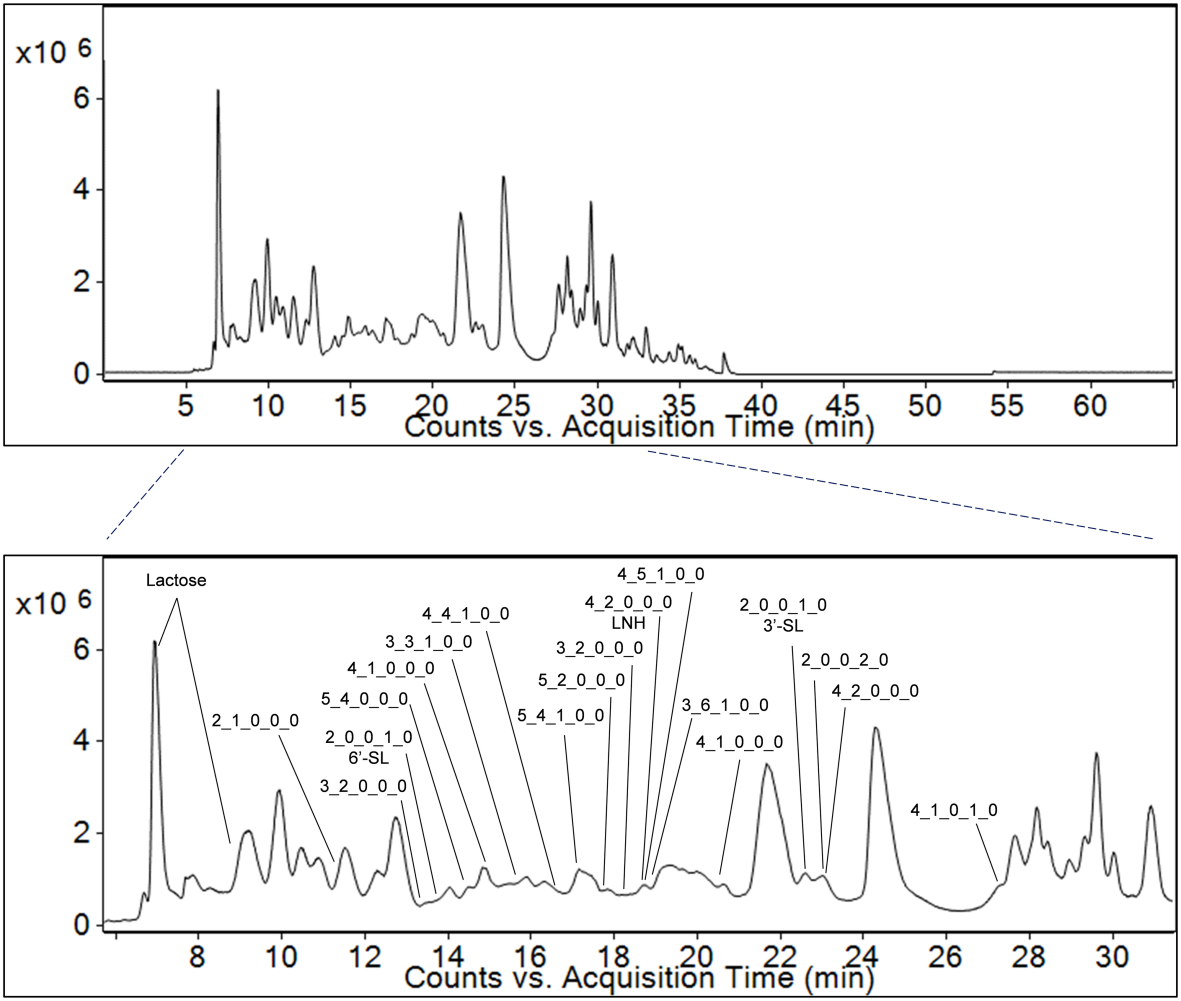
Annotated total ion chromatogram of aminoxyTMT-labeled bovine milk OS. OS are represented by their monosaccharide compositions, denoted as Hex_HexNAc_Fuc_NeuAc_NeuGc. 3’-SL, 3’-sialyllactose; 6’-SL, 6’-sialyllactose; LNH, lacto-N-hexaose.

Acidic (sialic acid-containing) OS compose the majority of bovine milk OS by concentration [38], and in this study we were able to identify and quantify the highly abundant 3’-SL, as well as 6’-SL and the OS with compositions 2 hexose (Hex) 2 N-acetylneuraminic acid (NeuAc) and 4 Hex 1 HexNAc 1 NeuAc. The OS profile of this sample included five fucose (Fuc)-containing OS and six neutral, non-fucosylated compounds. We observed the highly abundant neutral OS GalNAc(α1–3)Gal(β1–4)Glc characteristic of bovine milk [11, 31] (identity confirmed by analysis of available commercial standard), as well as OS with a relatively high degree of polymerization (DP), including those with compositions 5 Hex 2 HexNAc, 5 Hex 4 HexNAc, and the fucosylated 3 Hex 3 HexNAc 1 Fuc, 3 Hex 6 HexNAc 1 Fuc, 4 Hex 4 HexNAc 1 Fuc, 4 Hex 5 HexNAc 1 Fuc, and 5 Hex 4 HexNAc 1 Fuc. A total ion chromatogram of this milk sample with OS retention times annotated is shown as Fig 4.

**Fig 5:**
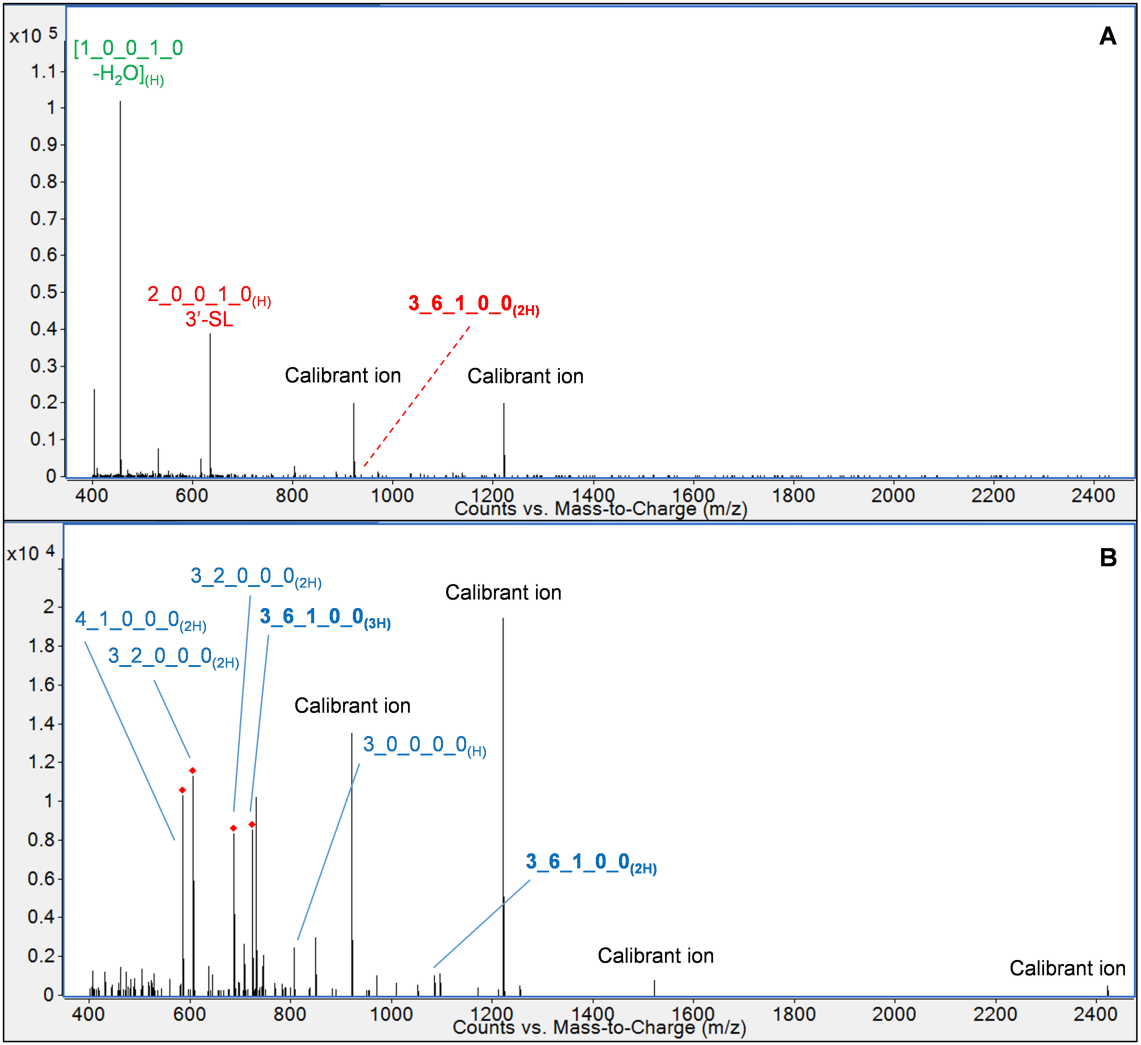
Ionization efficiency improvement following aminoxyTMT labeling. Injection of identical sample quantities before and after labeling resulted in increased signal intensity for numerous OS, as shown in the ESI mass spectra above for the OS of composition 3 Hex 6 HexNAc 1 Fuc (A–native OS; B–derivatized OS). Ions containing the aminoxyTMT label are annotated in blue; those without the label are shown in red. In-source fragments are annotated in green. OS are represented by their monosaccharide compositions, denoted as Hex_HexNAc_Fuc_NeuAc_NeuGc. 3’-SL, 3’-sialyllactose.

Compared to LC-MS analysis of native OS, labeling with the aminoxyTMT reagent provided a substantial increase in the ionization efficiency of the higher-DP analytes. Fig 5 shows mass spectra containing the OS with composition 3 Hex 6 HexNAc 1 Fuc, analyzed in the same sample both before and after derivatization. Labeling with the aminoxyTMT reagent increased the mass spectral peak height of the most abundant charge state by an approximate factor of 10, resulting in more consistent identification of this presumably low-abundance compound, as well as high-quality tandem spectra for quantification and confirmation of glycan compositions. As with other OS, derivatization increased the number of charges present on the most abundant precursor ion: in this case, the OS ionized as a 2+ ion in its native state but appeared most intensely as a 3+ ion following derivatization. In comparison to the drastic increase in signal intensity observed for larger OS, the ionization efficiencies of lower-DP OS were less affected by the derivatization.

**Fig 6:**
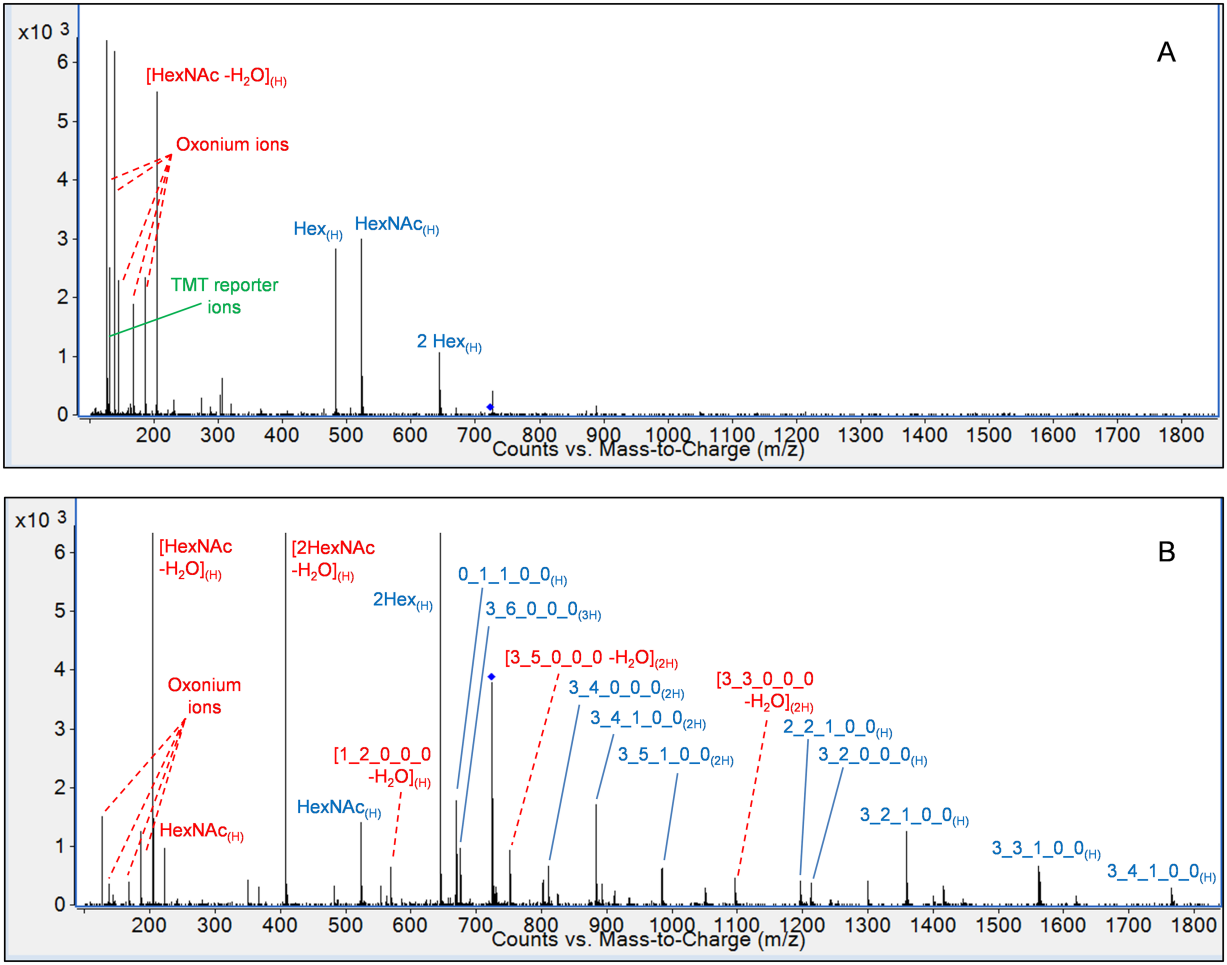
MS/MS spectra of the oligosaccharide with composition 3 Hex 6 HexNAc 1 Fuc. Two sets of collision energy parameters were developed: one for the purpose of relative quantification by aminoxyTMT reporter ions (A, CID 42.3V) and one for confirmation of monosaccharide composition (B, CID 7.3V). Fragments containing the aminoxyTMT label are annotated in blue; fragments without the label are shown in red. OS are represented by their monosaccharide compositions, denoted as Hex_HexNAc_Fuc_NeuAc_NeuGc.

Ideally, the same MS/MS spectra used for glycan quantification would also show sufficient glycan fragments to allow confirmation of OS monosaccharide compositions. We optimized collision energies to provide maximum reporter ion signal and ratio accuracy and found that the tandem spectra provided information on the monosaccharide composition of smaller glycans, but informative fragments were often missing for larger glycans. To resolve this issue, we implemented a second set of acquisition parameters that reduced collision energies by 16.39 V for 1+ ions, 6.75 V for 2+ ions, and 35 V for 3+ ions. These parameters were applied as necessary to confirm the presence of each OS in each sample set and provided much more comprehensive tandem spectra, which allowed the monosaccharide compositions of the OS to be confidently determined. Fig 6 provides a comparison of tandem spectra collected for the purposes of relative quantification (part A) and OS composition confirmation (part B). These findings are in agreement with a past observation that glycosidic bonds are broken more easily than the bond that release the TMT reporter ions [32].

### Comparison of fucosylated OS production between Holstein and Jersey cows

To further demonstrate the utility of this novel workflow, the relative abundance of high-molecular weight fucosylated OS were compared among milk samples from Holstein and Jersey dairy cattle. We measured the abundances of four fucosylated OS in a set of 20 milk samples, which represents the largest sample set analyzed by isobaric tagging in the current literature. Since the aminoxyTMT reagent set consists of six unique labels, past studies have limited their sample sizes to six or fewer samples when conducting relative glycan quantification. In order to expand the capability of the technique, we chose to multiplex the 20 samples into a set of four vials, with each vial containing an internal standard mixture that allowed glycan abundances to be normalized and compared among the four multiplexed sets.

**Fig 7:**
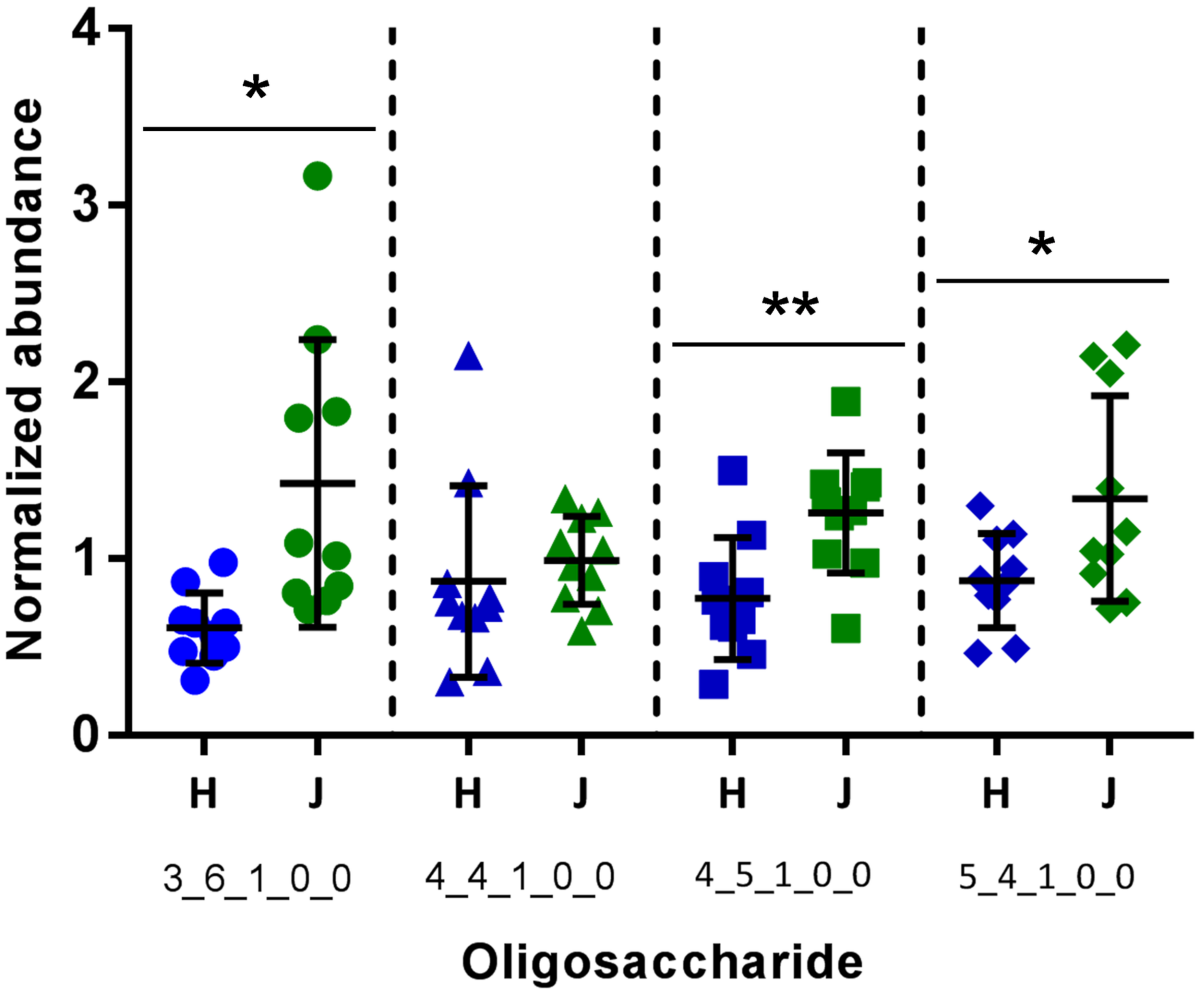
Comparison of four high-molecular weight fucosylated OS abundances in Holstein (H) and Jersey (J) milk. Error bars show mean ± standard deviation. OS compositions are expressed as Hex_NexNAc_Fuc_NeuAc_NeuGc. Asterisks represent statistical significance from a two-tailed unpaired t-test at p<0.05 (*) and p<0.01 (**).

Four fucosylated OS were identified in all 20 samples, with compositions 3 Hex 6 HexNAc 1 Fuc, 4 Hex 4 HexNAc 1 Fuc, 4 Hex 5 HexNAc 1 Fuc, and 5 Hex 4 HexNAc 1 Fuc. These specific compositions have been identified in two prior studies on bovine milk OS [11, 34]. Three of the OS (all except 4 Hex 4 HexNAc 1 Fuc) were significantly more abundant in the Jersey milk samples (Fig 7). Although significant differences were identified between the breeds, Fig 7 illustrates that the within-breed variations were also substantial, with some cows producing two to three times more of these OS than others.

## Discussion

One of the major barriers to success in the field of functional glycomics is the ability to readily characterize complex mixtures of OS in biological samples. Successful OS profiling often requires substantial concentration of the OS with concurrent removal of interfering matrix components, particularly those that will react with derivatizing agents. In the case of bovine milk, the lactose content must be substantially reduced prior to aminoxyTMT labeling and analysis to enable detection of the relatively trace OS. In this study, we present a method of extraction, derivatization, and analysis that successfully extends the application of relative quantification by isobaric labeling to free bovine milk OS. Separation of labeled and multiplexed OS on a PGC column followed by Q-TOF MS/MS analysis resulted in accurate quantification of 15 OS, including several larger OS that often give weak signals during MS analysis. The identified OS shown in Table 2 include OS that are known to have high relative abundances in bovine milk, including 3’-SL and the trisaccharide GalNAc(α1–3)Gal(β1–4)Glc [11, 31, 38]. The acidic bovine OS 3’-SL and 6’-SL are known to exist in human milk [40, 41] and are characterized for their ability to feed gut-related bacteria [42, 43]. The ability to monitor the abundance of these prebiotic compounds is a key objective in the design of novel analytical techniques that will guide development of functional dairy-based foods and ingredients. This method is able to measure quantities for the major classes of glycans in bovine milk, including acidic, neutral, and fucosylated OS. Although this method development was conducted with raw bovine milk, pasteurization does not affect the stability of the structurally similar OS in human milk [44], and therefore the technique should be applicable to pasteurized bovine milk as well without further modification. Furthermore, its ability to monitor low-abundance components of bovine milk may make this method suitable to the analysis of trace OS in other types of investigations, such as in glycomic profiling of blood and urine.

Isobaric labeling can provide improved data quality for specialized applications, in addition to the known advantages of minimizing differences in ionization between samples and shortening instrumental runtime. Past experiments have characterized the derivatization yield of the aminoxyTMT reagents as exceeding 95% [24], and recent publications have demonstrated their utility in the analysis of N-glycans [25, 32]. However, the fact that relative glycan quantities are derived from tandem-MS spectra implies that thorough method optimization is needed to ensure data reliability and accuracy. Furthermore, extension of the existing methods to new sample types requires careful testing and validation to identify unforeseen interferences associated with new sample matrices. Past publications utilizing isobaric labels for N-linked glycan analysis have noted compounds for which reporter ions are difficult to generate in sufficient abundance, presumably because larger glycans contain a greater number of bonds that fragment more easily than those that release the reporter ion, leading to a decreased likelihood of reporter ion formation [25, 32]. Strategies to overcome this complication include MS^3^ fragmentation of ions containing the intact tag [25] and generating more favorable charge adducts using a metal ion dopant to improve ion abundances [32]. Bovine milk OS, with a degree of polymerization of ≤10, are generally smaller than N-glycans, and we did not encounter difficulty generating sufficient reporter ion abundances by fragmenting OS-hydrogen ion adducts. We therefore avoided the use of metal ion dopants to provide simpler mass spectra. To obtain the best MS/MS spectra from these adducts, we developed two sets of collision energy parameters: one which used higher CID energies to generate maximum reporter ion abundances, and one with lower energies to provide spectra which confirmed the OS monosaccharide compositions (Fig 6).

The higher energy CID parameters allowed reliable relative quantification of 15 OS, with CVs among four replicate injections of extracted milk OS being below 15% for 11 of the 15 compounds. Of the OS with CVs above 15%, the most variable was 4 Hex 4 HexNAc 1 Fuc (CV 31.5%). This compound’s maximum peak height was near the 5,000 ion count fragmentation threshold and was only fragmented in two of the four injections, producing only two total MS/MS spectra. However, the accuracy of its average reporter ion ratio (2: 1.18) was still within the magnitude of error typically seen in isotopic ratio-based relative quantification studies [25, 32, 45], as was the case for the other OS with higher ratio CVs. Given that these ratio-based quantification techniques can typically quantify compounds with <20% error, they are well-suited to the comparison of biological samples in a variety of applications [32, 46, 47]. Studies requiring greater accuracy for the purposes of detecting minute differences between samples may be better suited to alternate analytical techniques, such as OS analysis by triple-quadrupole MS [48, 49]. However, this increased accuracy would come at the cost of reduced sample throughput due to the lack of multiplexing ability, and in the case of absolute quantification, would require the use of analytical standards that are currently unavailable for the majority of bovine milk OS.

We have theorized that allowing repeated MS/MS events for each analyte and averaging the reporter ion ratios is advantageous because it mitigates the effects of inevitable spectrum-by-spectrum ratio variations. Due to the relatively small number of compounds under examination in our study, we were able to disable in-run dynamic exclusion for tandem-MS fragmentation to collect these multiple MS/MS spectra without losing substantial opportunities to fragment lower-abundance OS. However, this approach may not be feasible in glycomic studies that aim to profile a larger number (>50) of analytes. Past studies have shown that, if needed, dynamic exclusion can be employed while still providing satisfactory data quality [25].

A particularly interesting finding was the substantial increase in ionization efficiency of the high-molecular weight fucosylated OS upon labeling (Fig 5), which allowed four of these compounds to be consistently identified in the sample set and represents a key advantage of the technique. Although two recent studies have identified subsets of these OS when profiling bovine milk [11, 34], other comprehensive studies employing similar profiling techniques have not found any fucosylated OS in mature bovine milk [31, 50]. We believe that this phenomenon is due to the apparently wide range of OS concentrations in milk, which results in a weak signal for lower-abundance OS when LC-MS injection quantities are optimized such that more abundant compounds do not cause detector saturation. The improved ionization efficiency afforded by the aminoxyTMT reagent has been noted in a recent study of N-glycan analysis [25], and in our case, the substantial improvement in low-abundance OS ionization decreased the disparity in signal strength among the pool of OS.

Fucosylated human milk OS, in addition to their prebiotic functions [51], can inhibit pathogen binding to intestinal cells [52] and are correlated with decreased incidence of diarrhea *in vivo* [5], making this class of compounds a potentially valuable dietary component. We found that the Jersey milk samples contained significantly higher average levels of three out of the four fucosylated compounds (Fig 7). Similar findings were published in a study by Sundekilde et. al., which found that Jersey milk contained higher levels of the fucosylated OS 3 Hex 6 HexNAc 1 Fuc and 4 Hex 5 HexNAc 1 Fuc [11]. Another noteworthy component of these results is the substantial within-breed variation in the abundance of these OS. This data suggests that while breed is likely a determinant of milk OS content, other within-breed or environmental factors could also substantially impact milk OS production. With the growing interest in developing infant foods that mimic the protective effects of human milk, as well as the fact that human milk OS are highly fucosylated [53], the fucose-containing OS in bovine milk and robust strategies for their analysis are likely to be of interest in future studies. We hope that this novel analytical technique will facilitate further characterization of fucosylated bovine milk OS and exploration of the within-breed factors influencing their abundance.

## Conclusions

Growing understanding of the relevance of free OS and protein-linked glycans to a multitude of biological processes and health outcomes is motivating development of techniques for their characterization and quantification. In particular, the significance of human milk OS for infant health and development is creating a need for strategies to provide industrial-scale quantities of these compounds in the marketplace. The availability of advanced analytical techniques will aid in developing products that mimic the content of human milk as closely as possible by allowing comprehensive profiling of similar OS available in dairy streams. The method developed in this study complements existing techniques by improving the reliability of profiling low-abundance compounds, as well as improving instrumental throughput for LC-MS analysis.

## Supporting information

S1 Fig Sample MS/MS spectra demonstrating normalization of sample reporter ion signals to a common internal standard. (TIF)

S2 Fig Sample MS/MS spectra of oligosaccharide standards used for initial method optimization. The aminoxyTMT reporter ion region (m/z 126–131) is shown as an inset at the right of each MS/MS spectrum. Each standard was labeled with the TMT^6^-126 and TMT^6^-127 reagents in a 2:1 molar ratio. A: 3’-sialyllactose, B: 6’-sialyllactose, C: Lacto-N-tetraose, D: Lacto-N-hexaose.
(TIF)

S1 Table Analysis of glycan standards labeled in 2:1 molar ratios with TMT6-126 and TMT6-127 reagents. (XLSX)

S2 Table Analysis of a bovine milk oligosaccharide extract labeled in a 2:1 molar ratio with TMT^6^-130 and TMT^6^-131 reagents—Injection #1. (XLSX)

S3 Table Analysis of a bovine milk oligosaccharide extract labeled in a 2:1 molar ratio with TMT^6^-130 and TMT^6^-131 reagents—Injection #2. (XLSX)

S4 Table Analysis of a bovine milk oligosaccharide extract labeled in a 2:1 molar ratio with TMT^6^-130 and TMT^6^-131 reagents—Injection #3. (XLSX)

S5 Table Analysis of a bovine milk oligosaccharide extract labeled in a 2:1 molar ratio with TMT^6^-130 and TMT^6^-131 reagents—Injection #4. (XLSX)

S6 Table Analysis of fucosylated oligosaccharide abundance in mid-lactation milk collected from Holstein (N = 10) and Jersey (N = 10) dairy cows. (XLSX)
