# Supplementary figures and images for: Multiplexed bovine milk oligosaccharide analysis with aminoxy tandem mass tags
Source: PLoS One. 2018 Apr 26;13(4):e0196513. doi: 10.1371/journal.pone.0196513 (PMC5919578; doi:10.1371/journal.pone.0196513)

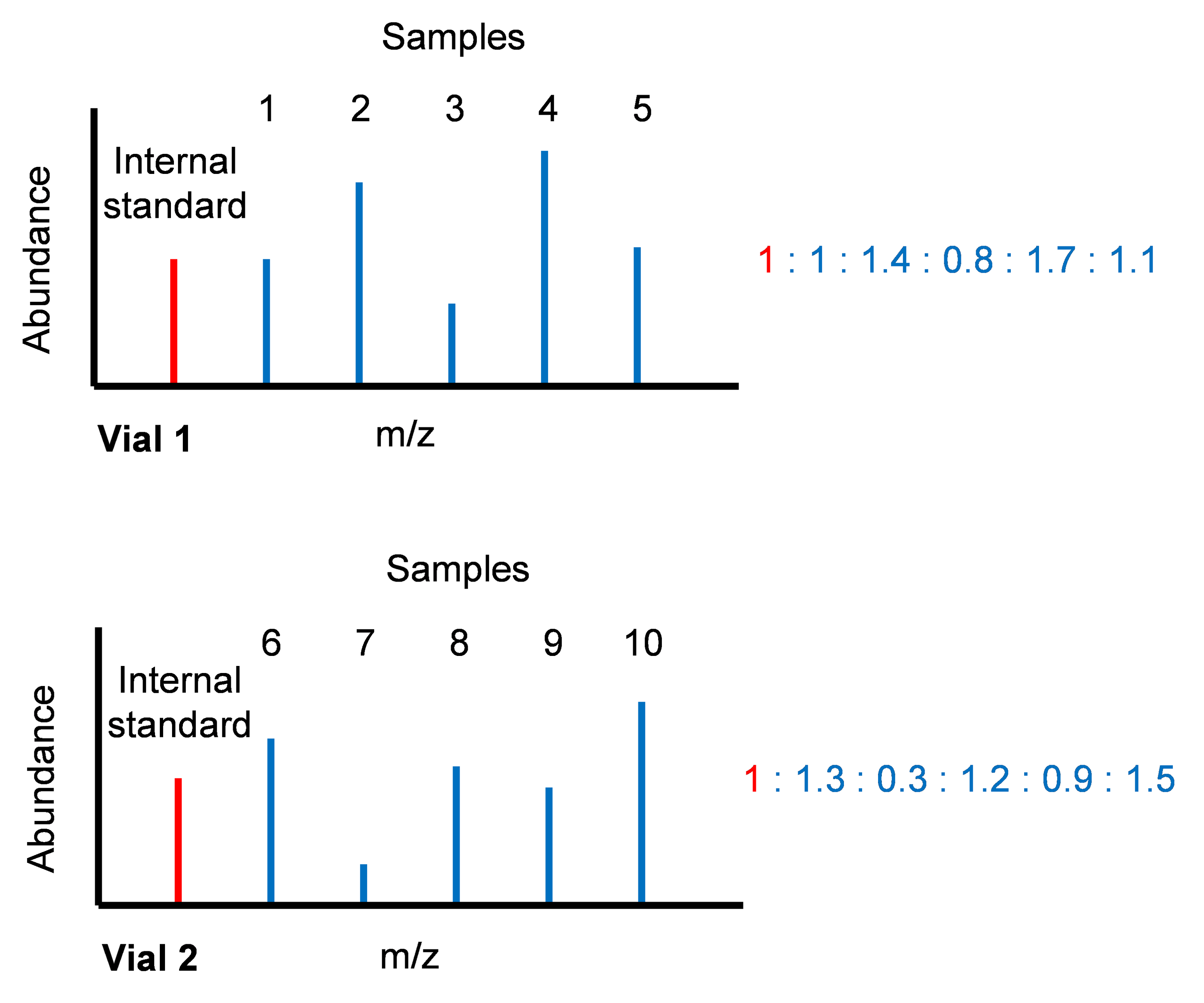

Supplement: S1 Fig — (TIF) [file pone.0196513.s001.tif]

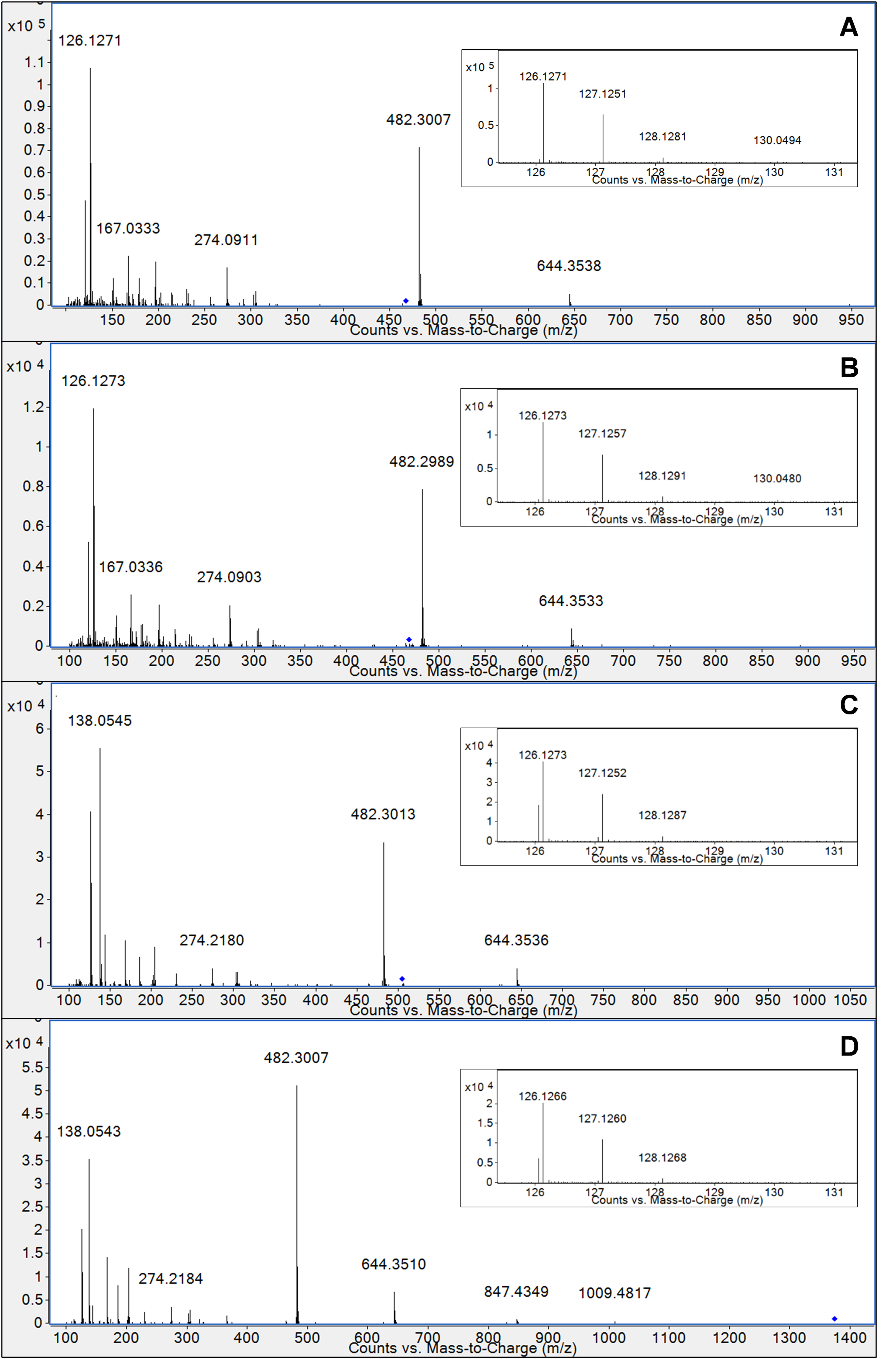

Supplement: S2 Fig — The aminoxyTMT reporter ion region (m/z 126–131) is shown as an inset at the right of each MS/MS spectrum. Each standard was labeled with the TMT6-126 and TMT6-127 reagents in a 2:1 molar ratio. A: 3’-sialyllactose, B: 6’-sialyllactose, C: Lacto-N-tetraose, D: Lacto-N-hexaose. (TIF) [file pone.0196513.s002.tif]
